# Second-Generation Dual Scan Mammoscope With Photoacoustic, Ultrasound, and Elastographic Imaging Capabilities

**DOI:** 10.3389/fonc.2021.779071

**Published:** 2021-11-19

**Authors:** Emily Zheng, Huijuan Zhang, Soumya Goswami, Irteza Enan Kabir, Marvin M. Doyley, Jun Xia

**Affiliations:** ^1^ Department of Biomedical Engineering, University at Buffalo, The State University of New York, Buffalo, NY, United States; ^2^ Department of Electrical and Computer Engineering, Rochester Center for Biomedical Ultrasound, University of Rochester, Rochester, NY, United States

**Keywords:** photoacoustic (PA), photoacoustic (optoacoustic) imaging, breast (diagnostic), breast cancer, imaging, ultrasound, elastography, dual scan mammoscope

## Abstract

We recently developed the photoacoustic dual-scan mammoscope (DSM), a system that images the patient in standing pose analog to X-ray mammography. The system simultaneously acquires three-dimensional photoacoustic and ultrasound (US) images of the mildly compressed breast. Here, we describe a second-generation DSM (DSM-2) system that offers a larger field of view, better system stability, higher ultrasound imaging quality, and the ability to quantify tissue mechanical properties. In the new system, we doubled the field of view through laterally shifted round-trip scanning. This new design allows coverage of the entire breast tissue. We also adapted precisely machined holders for the transducer-fiber bundle sets. The new holder increased the mechanical stability and facilitated image registration from the top and bottom scanners. The quality of the US image is improved by increasing the firing voltage and the number of firing angles. Finally, we incorporated quasi-static ultrasound elastography to allow comprehensive characterization of breast tissue. The performance of the new system was demonstrated through *in vivo* human imaging experiments. The experimental results confirmed the capability of the DSM-2 system as a powerful tool for breast imaging.

## Introduction

Breast cancer is caused by mutations and abnormal amplification of associated genes. Uncontrolled growth of breast cells from malignant tumors will further invade the surrounding tissue and threaten the patient’s life (1). Several clinical trials demonstrate that early detection is crucial for improving the survival rate of patients (1). Based on the variations in tissue compositions, breast tissue can be classified into four categories: Group A (almost entirely fatty), Group B (scattered areas of fibroglandular density), Group C (heterogeneously dense), and Group D (extremely dense), corresponding to an increasing percentage of dense tissue in the breast (2): The breast tissue mainly consists of fat, and is associated with the lowest risk of breast cancer. Groups B and C are the intermediate levels. Group D indicates the highest percentage of dense tissue. People who have been classified into this category are the most susceptible to breast cancer (2). X-ray mammography is the most widely used method for breast cancer detection. However, because epithelial and connective tissues appear white on the mammogram images, dense breast leads to the superimposition of overlapping radiopaque breast tissues. As a result, the mammogram shows an inverse relationship between system sensitivity and breast tissue density (3). Other breast imaging modalities, such as magnetic resonance imaging (MRI) and ultrasound (US), also have their strengths and limitations in breast imaging: MRI exhibits high spatial resolution and sensitivity even for dense breasts (4) and can maintain a specificity above 86% (5). However, the injection of gadolinium as a biomarker is associated with an increased risk of allergy and kidney damage (6, 7). US system is cost-effective and radiation-free; however, it suffers from a high false-positive rate and operator error (8). Therefore, US is mostly considered as a supplemental screening tool (9). Hence, a safe and effective modality for the screening of patients with high breast density is emergently needed.

Photoacoustic (PA) is an emerging imaging technology that combines the high contrast of optical imaging and the high resolution of ultrasonic imaging (10). PA technique use diffused light as a source of optical irradiation. Light-induced heat leads to thermoelastic expansion in the target tissue, generates pressure, and emits ultrasonic waves. The resulting ultrasonic waves, referred to as PA signals, are then detected by the ultrasonic transducer. The reconstructed image shows the initial pressure distribution to reveal the absorbing structures in the targeted tissue (11). Overall, photoacoustic tomography (PAT) provides a radiation-free approach to image the breast with high optical contrast and high spatial resolution. These characteristics allow PAT to potentially fulfill the clinical gap in current breast imaging techniques (12). Because different biological chromophores (hemoglobin, water, lipid, etc.) have different absorption spectra, they can be differentiated in PAT using different incident wavelengths (13). Hemoglobin is the most widely used contrast for photoacoustic imaging of the breast (14). The formation of breast tumors is typically associated with additional feeding vessels, resulting in increased regional vascularity and higher local hemoglobin level (15). By mapping the hemoglobin distribution and concentration in the breast, PAT offers non-invasive, label-free imaging of breasts tumor. A review of photoacoustic features of breast cancer can be found in recent literatures (14, 16, 17).

We recently described a first-generation dual-scan mammoscope (DSM-1) system. Similar to mammogram, DSM-1 images patients in the standing pose. However, the compression was achieved by two plastic films, thus was very mild (18). Scanning is performed by the transducer sets placed inside the water tanks in a co-planar configuration. During imaging, the setup operates along the craniocaudal (CC) plane, illuminating the breast from both the top and bottom sides of the tissue. The system utilizes an Nd : YAG laser with 1064nm output, which provides the optimum balance between hemoglobin absorption and imaging depth. The PA signals are detected by two 128-element linear transducers. Both transducers have a central frequency of 2.25 MHz. After data acquisition, we reconstruct the image acquired by each transducer separately and then combine them to form the final 3D image. Our previous results have demonstrated that the DSM-1 system has a spatial resolution of approximately 1mm along all directions and can image through 7cm of breast tissue (18).

However, despite being a portable system with high spatial resolution, the DSM-1 system has several limitations. First, the lateral length of the transducer array (85.6mm) cannot cover the entire breast. Second, the system was constructed with optical posts with large freedom in position alignments, which caused variations in the transducer position between experiments. Therefore, an additional step of system calibration is required in the image reconstruction process. Finally, because of the low-quality US images, cross-validating data with the clinical US becomes challenging.

Compared with the previous system, DSM-2 contains a precisely machined system setup. The improved system stability minimizes the variation in transducer positioning and allows us to standardize the image reconstruction procedure. We also introduced a laterally shifted round-trip scanning scheme, increasing the lateral coverage from 85.6 mm to 171.2 mm, which is sufficient to cover the entire breast. Finally, using a motorized compression control system, DSM-2 could perform quasi-static compression, allowing us to use tissue stiffness as an additional feature of tumor characterization.

## Methods

### Compression Control System

**Figure 1:**
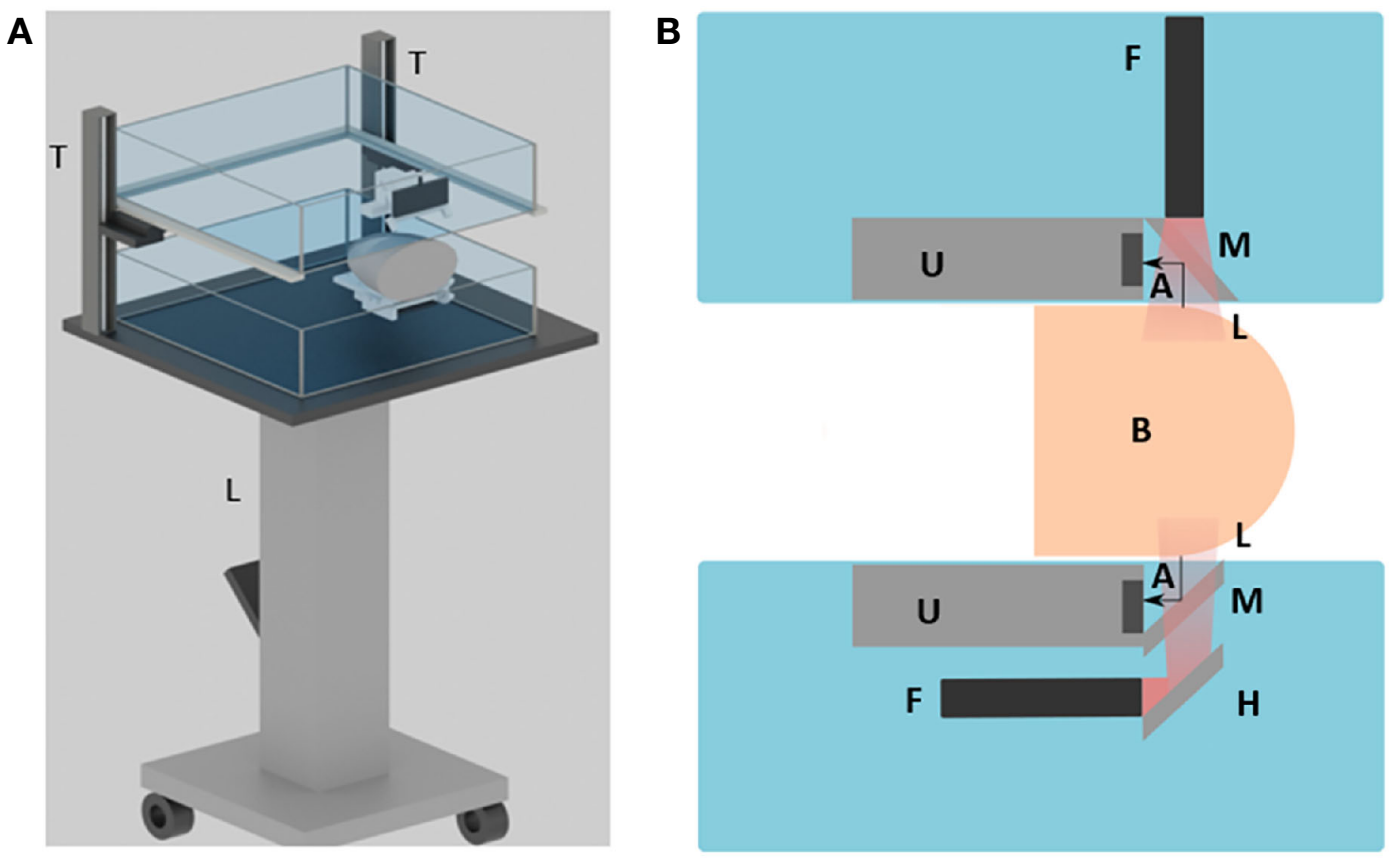
Overview of the DSM-2 system setup. **(A)** 3D schematic drawing of the system. L, the lift table; T, translation stages. **(B)** Cross-sectional view of the system. A, acoustic wave; F, fiber bundle; H, hot mirror; L, light beam; M, cold mirror; and U, the ultrasound transducer array.

DSM-2 is derived from the previous DSM-1 system; thus, they share the same fundamental mechanical setting. The system is built on a foot-operated mobile lift table (McMASTER-Carr, Model No. 24485T22) with adjustable height. As shown in **Figure 1A**, one water tank (bottom) was fixed on the optical breadboard, and the other water tank (top) was mounted on two vertically placed 20cm motorized translation stages (McMASTER-Carr). This is an improvement over the original system, which used manual positioning through gear rack posts. In this new design, we can program the translation stages to adjust the height of the water tank and perform compression. By setting the frequency and the number of the pulses sent to the motorized stage, we can precisely control the speed, distance, and direction of water tank movement, enabling quasi-static US elastography.

### Transducer and Fiber Output Combiner

For PA signal generation and detection, we couple two 128-element linear transducers with a bifurcated line-output fiber bundle. During scanning, each transducer-fiber bundle set is immersed into one of the water tanks. The breast tissue is mildly compressed by the water tanks for imaging (**Figure 1A**). For the top transducer, a high-performance cold mirror (TECHSPEC cold mirror, Edmund Optics Inc.) is attached to the transducer at 45° (**Figure 1B**). The cold mirror allows for 97% of 1064 nm light to pass through at a 45° incident angle During imaging, the light beam is first emitted from the fiber bundle, and then passes through the cold mirror to perform illumination. The generated PA signals are reflected by 90° toward the transducer by the cold mirror for data acquisition. For the bottom set, we adapt a double-mirror design instead of a single reflector to save space, as shown in **Figure 1B**. Compared with the single-reflector design, we added a high-performance hot mirror for the bottom side. The hot mirror will reflect the light beam by 90° toward the cold mirror. After the light beam passes through the cold mirror, the PA signal then gets generated and received in the same way as from the top side.

**Figure 2:**
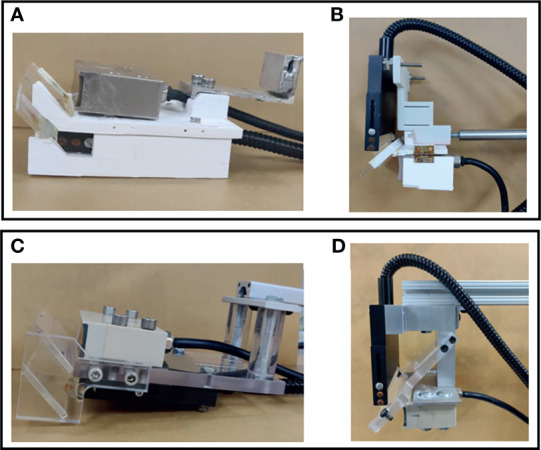
Design of the transducer-fiber combiner. The first row shows photos from the DSM-1 system, and the second row shows the photos from the DSM-2 system. **(A)** 3D-printed bottom transducer-fiber set. **(B)** 3D-printed top transducer-fiber set. **(C)** Precisely machined aluminum-acrylic bottom transducer-fiber set. **(D)** Precisely machined aluminum-acrylic top transducer-fiber set.

In DSM-2, we precisely machined the transducer and fiber combiner instead of 3D printing them, which was done in the first generation. Compared with the 3D printed holder (**Figures 2A, B**), the new setting (**Figures 2C, D**) is more advanced in several aspects. First, the new transducer-set holders can ensure a 45° attachment of mirrors, can optimize the passage of light and can increase the intensity of the acquired PA signal. Second, the new system is made with aluminum and acrylic, which will last longer than the polylactic acid (PLA) material used in 3D printing. Finally, the new mount allows quick connection with T-slot positioning bars, offering instant and reproducible positioning of the scanning head and facilitating calibration-free scanning.

In both DSM systems, we must combine data from both transducers to form a complete image of the breast. In the DSM-1 system, two transducer sets need to be manually aligned before each experiment to ensure maximum overlap of the scanning region. In addition, after each scanning, we need to perform a calibration scan with a phantom to determine the spatial offset between two transducer sets. In DSM-2, optical post assemblies are replaced with T-slot framing to provide more robust support and fast assembly. With improved mechanical stability, we can standardize the offset for image combination, and the calibration steps are no longer required.

### Field of View

The field of view of the DSM-1 system was limited by the active width of the transducer (85.6 mm). In DSM-2, we increased the lateral coverage through a laterally shifted round trip scan.

**Figure 3:**
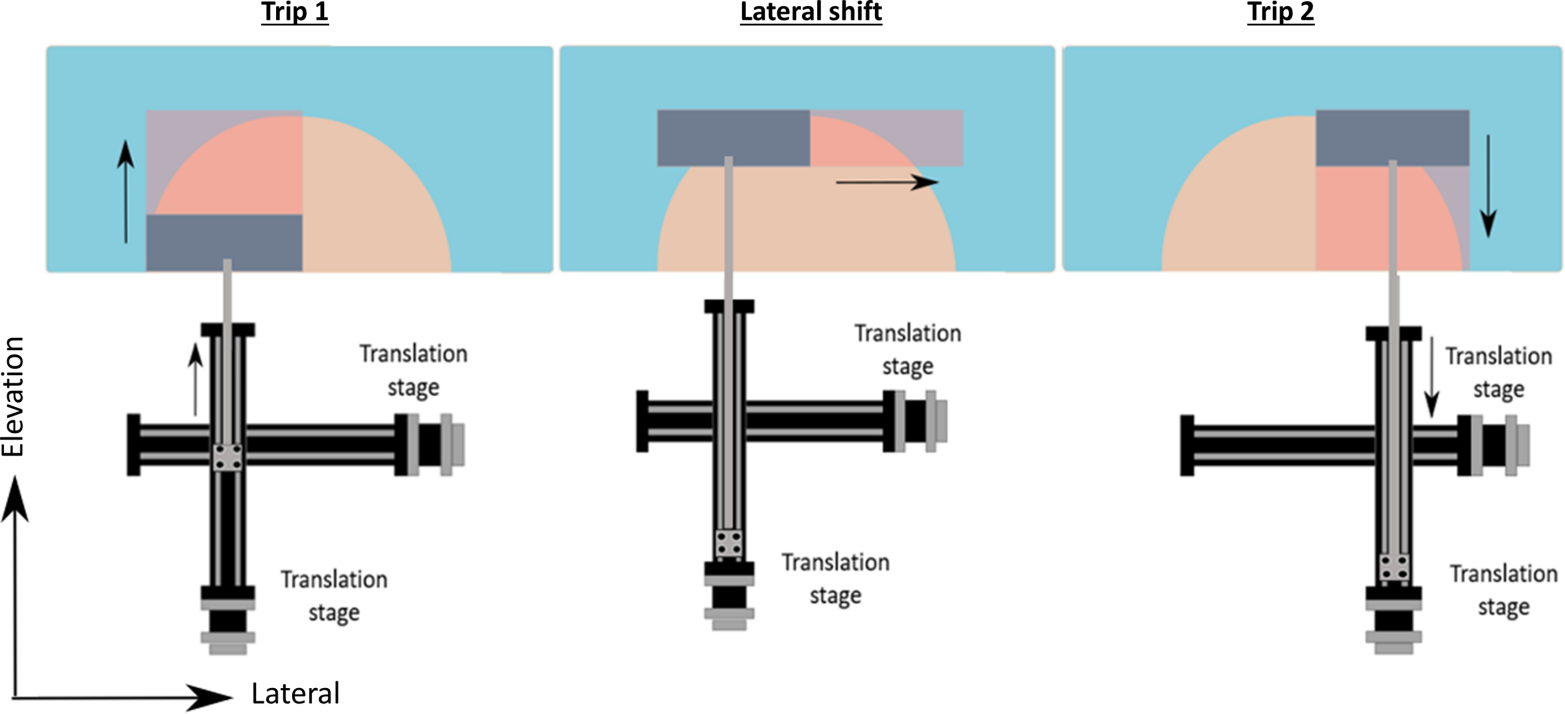
Schematic drawing of the round-trip scanning. Trip 1: the transducer is placed alone the left side of the breast and scans away from the chest wall; Lateral shift: lateral movement of 85.6 mm; Trip 2: the transducer scans toward the chest wall to cover the right half of the breast.

The DSM-2 system consists of two translation stages placed perpendicular to each other, which perform round-trip scanning. The process of round-trip scanning can be divided into three steps, as shown in **Figure 3**. First, we place the transducer at the left portion of the breast and scan away from the chest wall (trip 1). Second, the movement switches to the second translation stage to perform a lateral shift of 85.6 mm. This lateral offset was set to be the active width of the transducer to facilitate accurate image overlapping. After shifting, the transducer sets now cover the right portion of the breast tissue. Finally, the transducer was scanned toward the chest wall to complete imaging (trip 2). Based on reference (19), the average value of medial mammary radius (MR) and lateral mammary radius (LR) among 385 females were 88 and 80 mm, respectively. By adding MR and LR, we estimated the average breast width diameter to be 168 mm. The combined scan protocol can provide a lateral coverage of 171.2 mm, which allows us to cover the whole breast tissue in most cases. To compensate for the increased time in round trip scanning, we increased the step size of the motors from 0.1 to 0.5 mm/pulse. Because the system has a spatial resolution of ~1 mm along the scanning direction (18), the 0.5 mm step size won’t affect the image resolution. For DSM-2, the total scanning time for a single breast is approximately 40 seconds.

### Quasi-Static Elastography

In the DSM-2 system, we incorporated US elastography to visualize the mechanical properties of the breast. Previous studies have indicated that compared with benign lesions, malignant breast masses are typically stiffer than the surrounding healthy tissue (20). Thus, elastography is an effective imaging modality for detecting breast abnormalities. In addition, elastography can also characterize other biomechanical properties, including shear modulus (21), tissue nonlinearity (20), and viscosity (22). Therefore, the addition of elastography will allow us to perform a comprehensive characterization of breast tissue.

#### Experimental Setup

In DSM-2, we implemented quasi-static US elastography using the two water tanks shown in **Figure 1A** as the compression system. Utilizing motorized control, we can precisely control the amount of compression in our experiments. Before compression, we first acquire a control image with plane-wave imaging through four different angles between -4˚ and 4˚ (23). Then, we applied a 1% uniaxial compression by lowering the top water tank. Ultrasound data were then acquired with the same plane wave imaging method. Delay-and-sum beamforming implemented in a graphics processing unit (GPU) was applied to the raw channel data.

#### Phantom

We have performed a phantom experiment to validate the performance of US elastography. Homogeneous tissue-mimicking gelatin phantoms were fabricated from 200-bloom type A (Custom Collagen, Addison, IL) gelatin. The phantoms had a volume of 800 ml, with 8% gelatin concentration, 1% cornstarch for US imaging, and the remaining volume of deionized (DI) water. Cylindrical inclusion phantoms were fabricated with an inclusion diameter of 1.5 cm. The inclusion gel solution had a higher gelatin concentration (15%) than the background (8%) to make the inclusion stiffness higher compared with the background. Furthermore, 3% cornstarch in volume was used for the inclusion solution to increase the ultrasound echogenicity. First, a gelatin solution was prepared for the background medium was poured in a mold containing a 1.5 cm diameter plastic cylinder, and was allowed to solidify. The cylinder was then removed, and the gelatin solution for the inclusion medium was poured into the cylindrical space and allowed to solidify.

#### Strain Mapping With 2D Motion Registration

The axial (dz) and lateral (dx) displacements between the pre and post-compressed RF echo frames were estimated using a 2-D cross-correlation-based similarity search algorithm (21, 24). A 2.5 mm × 2.5 mm kernel was applied to track motion between the pre-and post-compressed echo frames with an overlap of 80% in both the axial and lateral directions. This kernel size was computed by taking 15 channel lines of RF data laterally and 7–10 wavelengths of each channel line of RF data axially. We used 2-D spline interpolation to calculate the subpixel displacements (24). The displacement images were then used in a model-based inverse approach for elastic modulus estimation (25)—a process called quasi-static elastography.

## Results

### US B Mode Acquisition

**Figure 4:**
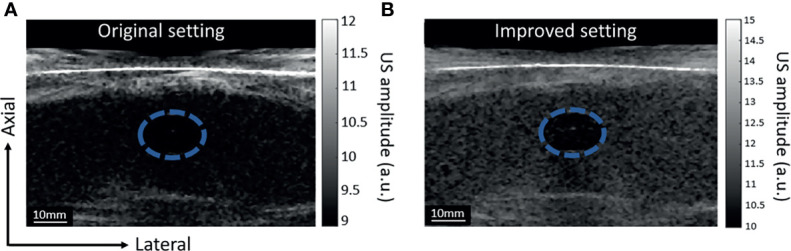
Grayscale US images of the phantom with inclusion, images acquired with two different settings. The blue-dashed circle highlights the location of the inclusion. The difference in corn starch concentration generates contraction in US images. **(A)** US image acquired with the DSM-1 setting: 5 incident angles and US acquisition voltage of 1.6 V. **(B)** US image acquired with the DSM-2 setting: 9 incident angles and US acquisition voltage setting of 10 V.

In the DSM-2, we improved the quality of the US image by increasing the number of incident angles from 5 to 9 and the transmit voltage from 1 V to 10 V. To quantify the improvement, we tested the new US signal acquisition setting with a phantom experiment. The phantom comprised a hypoechoic inclusion with 16% agar and 3% corn starch embedded in a base of 8% agar and 1% corn starch. During the experiment, the phantom was placed under the water tank for compression, and a single transducer array was used for scanning. We repeated scanning twice with two sets of parameters: the original setting (5 incident angles, 1.6 V firing voltage), and the improved version (9 incident angles, 10 V firing voltage) for comparison. The results are displayed in **Figures 4A, B**, respectively. The new US acquisition setting provided a greater contrast-to-noise ratio (CNR) and contrast ratio (**Table 1**).

**Table 1:** Contrast-to-noise ratio (CNR) and contrast ratio (CR) in the US images acquired by the DSM-1 and DSM-2 settings.

Setting	CNR (dB)	Contrast (dB)
DSM-1	6.6	6.1
DSM-2	15.9	28.2

### Quasi-Static Elastography

**Figure 5:**
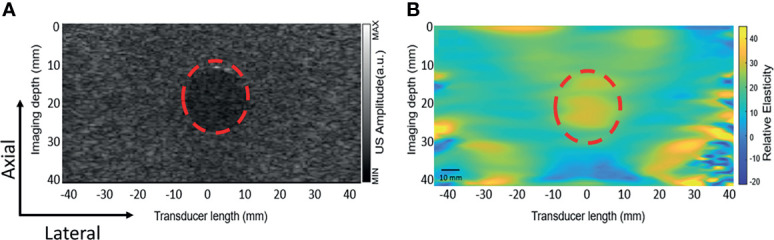
Elastography result obtained from the gelatin phantom. The red dashed circle highlights a cylindrical inclusion with a different elasticity than the surrounding medium. **(A)** The ultrasound B mode image. **(B)** The elastic modulus map obtained by uniaxial quasi-static compression.

**Figure 5A** shows the B-mode image and the corresponding tissue elastic modulus image acquired by the DSM-2 system. The modulus map is shown in **Figure 5B**, which reflects the inclusion structure in the center of the image, with values of 35 ± 6 kPa. These values suggest that the DSM-2 system can differentiate among tissues with different elasticities for breast cancer screening.

### Laterally Shifted Round-Trip Scanning

**Figure 6:**
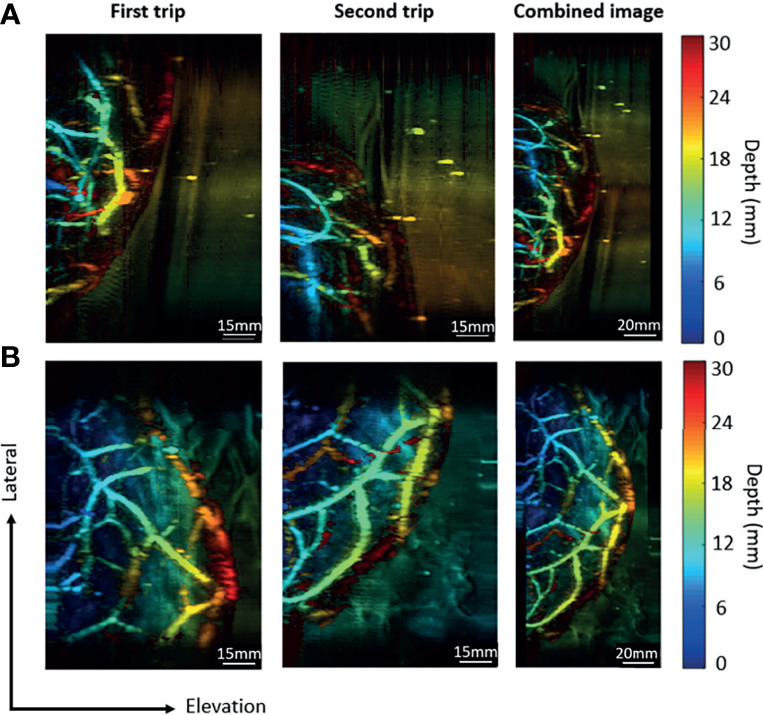
Combination of images acquired in the round-trip scan. **(A)** The imaging results for a breast cup size of C. **(B)** The imaging results for a breast cup size of D. The first trip covers the right portion of the breast, while the second trip covers the left portion of the breast. The last column contains combined images from two trips.

To quantify the performance of the new imaging modality, we imaged two volunteers with breast cup sizes C and D for system testing. The distances of the lateral shift were 85.6 mm in trial (a) and 60.3 mm in the trial (b). The thicknesses of the breast tissue after compression were 4 cm and 4.5 cm, respectively, due to the difference in cup size. After image reconstruction, we presented the data in depth-encoded maximum intensity projection (MIP) images, with the color scale from blue to red representing shallow to deep regions. The first two columns in **Figure 6** show the images acquired during each trip for both volunteers, each with an imaging area of 85.6 mm and 70 mm along the lateral and elevation directions, respectively. To combine the two images, we shifted the image of the second trip by the corresponding distance of lateral shift. The third row in **Figure 6** shows the combined image for both trials, with a total FoV of 171.2 mm along the lateral direction for trial (a) and 145.9 mm for trial (b).

### Combination of Vascular Signals Acquired by the Top and Bottom Transducers

**Figure 7:**
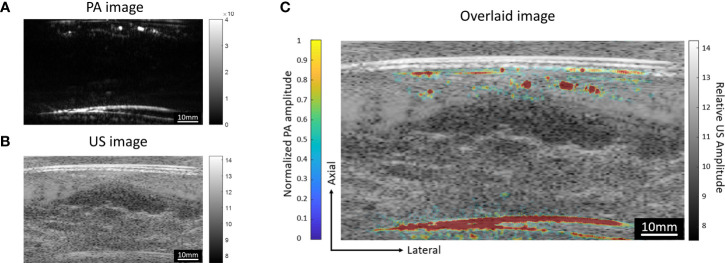
Cross-sectional **(A)** PA and **(B)** US images acquired by the top transducer. Both images show the strong interfaces created by the breast surfaces. In the PA image, depth-enhanced weighting was removed to reveal the signals of the breast surfaces. **(C)** PA image overlaid on the US image. The US image is shown in grayscale, while the PA image is shown in color.

The round-trip scans form two MIP images, one for the top transducer and the other for the bottom transducer. To obtain the final image of the whole breast, we need to combine these images. The lateral and elevation (scanning direction) offsets of the two transducers were fixed and calibrated through a phantom experiment. The phantom was made with two crossing pencil leads embedded in a ballistic gel. After reconstructing the top and bottom MIP images, we used 2D cross-correlation to find the shift between the two images. Our result indicates that the lateral shift is 2.4 mm and the elevational shift is 2.6 mm. While the lateral and elevation offsets of the two transducers are fixed, the axial distance between two transducers still varies among patients. To calculate the axial distance between two transducers, we utilize either PA or US images because both could reveal the top and the bottom boundaries of the breast surfaces. **Figures 7A, B** show the cross-sectional PA and US images acquired by the top transducer. Both images show the two strongest interfaces created by the top and bottom breast boundaries. We calculate the area of PA reconstruction and then overlap the estimated region upon the boundary signals. By performing the same operation for the bottom transducers, we can align the boundaries in the top and bottom images to quantify the axial distance between two transducers. For instance, for the subject data shown in **Figure 7**, we quantified the axial distance between two transducers to be 107 mm.

**Figure 8:**
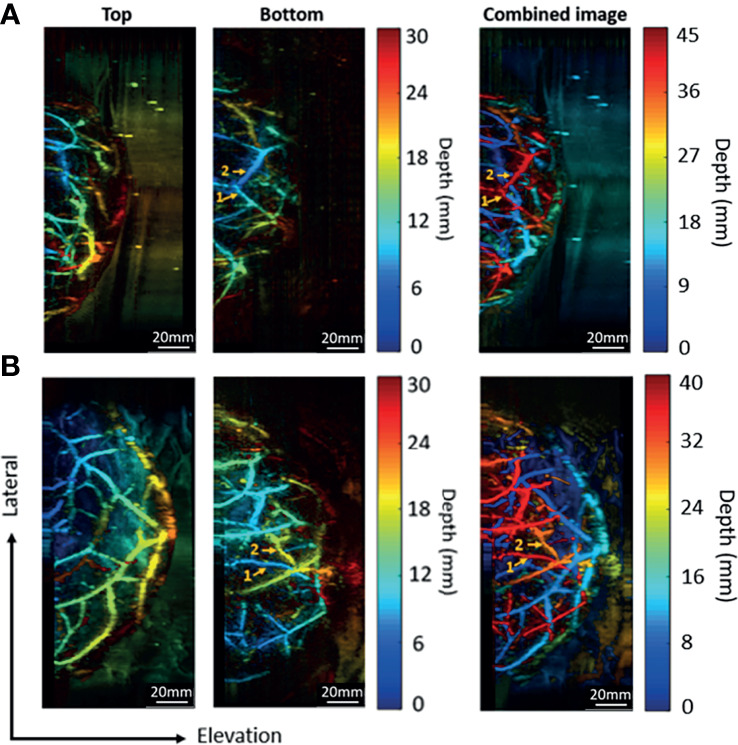
Combination of images acquired by the top and bottom transducers. **(A)** The imaging results for a breast cup size of C. **(B)** The imaging results for a breast cup size of D. The left two columns show the images acquired by the top and the bottom transducers, respectively. The right column shows the combined image.

Since the two transducers perform signal acquisition from the opposite directions, the data acquired from the bottom side will be flipped along the axial direction before the combination. In **Figure 8**, for both two trials, the same vessels acquired by the bottom transducer are marked with the same numbers. It can be noticed that the vessels look blue in the bottom transducer acquired image (close to the bottom transducer) turned red in the combined images (far away from the top transducer) after combination. The final combined images show the overall distribution of vascular structure in a top-down perspective.

## Discussion

We have developed the second-generation DSM system and tested its performance in human subjects. Compared to the previous version, DSM-2 is more advanced in several aspects, as summarized in **Table 2**. In terms of mechanical construction, DSM-2 overcomes the limitation of the transducer width by introducing a lateral shift during scanning. With the round-trip scanning system and increased step size, the new scan protocol doubles the field of view along the lateral direction, without sacrificing the image acquisition time: the DSM-1 system requires 50 seconds to scan an area of 50 × 80.6 mm along the lateral and elevational directions; in contrast, DSM-2 takes only 38 seconds to cover an imaging area of 70 × 171.2 mm. In addition, 3D-printed fiber-transducer combiners and optical posts are replaced with precisely-machined aluminum pieces and T-slot structures. The improved mechanical design minimizes the variation in system alignment and facilitates the image combination for round-trip imaging modality.

**Table 2:** Comparison of the system parameters between the first-generation and the second-generation DSM.

Parameter	DSM-1	DSM-2
Compression system	Manual	Motorized
Transducer-fiber combiner	3D printed	Precisely machined aluminum and acrylic
System setup	Optical post	T-slot framing
Lateral coverage	85.6mm	171.2mm
US acquisition	5 incident angles, acquisition voltage of 1.6V	9 incident angles, acquisition voltage of 10V
Scanning time	50 seconds	38 seconds
Quasi-static elastography	Not available	Available

Our previous study in DSM1 indicated that the quality of the US image was poorer in comparison to the clinical ultrasound machine (26). In DSM-2, we improved the US image quality by increasing the firing voltage and the number of firing angles in plane-wave US image acquisition. Based on the resulting B mode images obtained from the phantom experiment, the new acquisition parameters showed two times improvement in CNR and over four times improvement in the image contrast. These improvements will allow DSM-2 to better quantify the structure of breast tissue.

Based on the literature, the contraction of collagen generates extra stiffness in malignant tumors compared to normal tissue (27). Therefore, we also implemented quasi-static elastography in DSM-2. To evaluate the performance of ultrasound elastography, we performed an experiment with tissue-mimicking gelatin phantom. Compression was performed through the motorized compression control system. Our results indicate that DSM-2 can differentiate compositions based on their elasticity by mapping the strain across the imaged region. The adaption of US elastography will allow us to utilize tissue stiffness as an additional feature to identify malignancy, assist in tumor classification, and reduce the number of unnecessary biopsies (28).

## Conclusion

In this study, we introduced the DSM-2 system with various improvements over the first-generation system. Through phantom and human subject experiments, we confirmed that the second-generation system dramatically increased the lateral coverage of the system without sacrificing the scanning time or imaging resolution. Moreover, with improved mechanical stability, we obtained a standardized image reconstruction procedure, which allowed us to simplify the image reconstruction procedure and ensure a more accurate image combination. US image acquisition was also improved to achieve better image quality. The motorized compression system allows the system to apply quasi-static compression and facilitates the implementation of quasi-static elastography. Elastography is a new imaging modality that can aid in the functional characterization of the tumors by visualizing the stiffness variation within the ultrasonic scanning region. In future studies, we will test the DSM2 system in patients with breast cancer to further test the system performance.

## Data Availability Statement

The raw data supporting the conclusions of this article will be made available by the authors upon request.

## Ethics Statement

The studies involving human participants were reviewed and approved by University at Buffalo IRB committee. The patients/participants provided their written informed consent to participate in this study.

## Author Contributions

EZ designed the transducer-fiber holders, constructed the motorized compression control system, built the LabView code to conduct round-trip scanning, tested out the new US acquisition parameters, and performed volunteer experiments to evaluate the performance of the DSM-2 system. HZ performed phantom experiments to verify the performance of the quasi-static elastography imaging modality, and assisted the volunteer experiments. SG and EK provided technical assistance about the implementation of the quasi-static elastography modality. JX and MD supervised the study. All authors contributed to the article and approved the submitted version.

## Funding

This work was supported by a grant from the National Institutes of Health (R01EB029596).

## Conflict of Interest

JX is the founder of Sonioptix.

The remaining authors declare that the research was conducted in the absence of any commercial or financial relationships that could be construed as a potential conflict of interest.

## Publisher’s Note

All claims expressed in this article are solely those of the authors and do not necessarily represent those of their affiliated organizations, or those of the publisher, the editors and the reviewers. Any product that may be evaluated in this article, or claim that may be made by its manufacturer, is not guaranteed or endorsed by the publisher.
